# Supplementary material for: Allelic gene conversion softens selective sweeps
Source: bioRxiv. 2023 Dec 5:2023.12.05.570141. Preprint. [Version 1] doi: 10.1101/2023.12.05.570141 (PMC10723294; doi:10.1101/2023.12.05.570141)
Supplement: 1 [file NIHPP2023.12.05.570141V1-supplement-1.pdf]

bioRxiv preprint doi: https://doi.org/10.1101/2023.12.05.570141; this version posted December 5, 2023. The copyright holder for this preprint (which was not certified by peer review) is the author/funder, who has granted bioRxiv a license to display the preprint in perpetuity. It is made available under aCC-BY 4.0 International license.

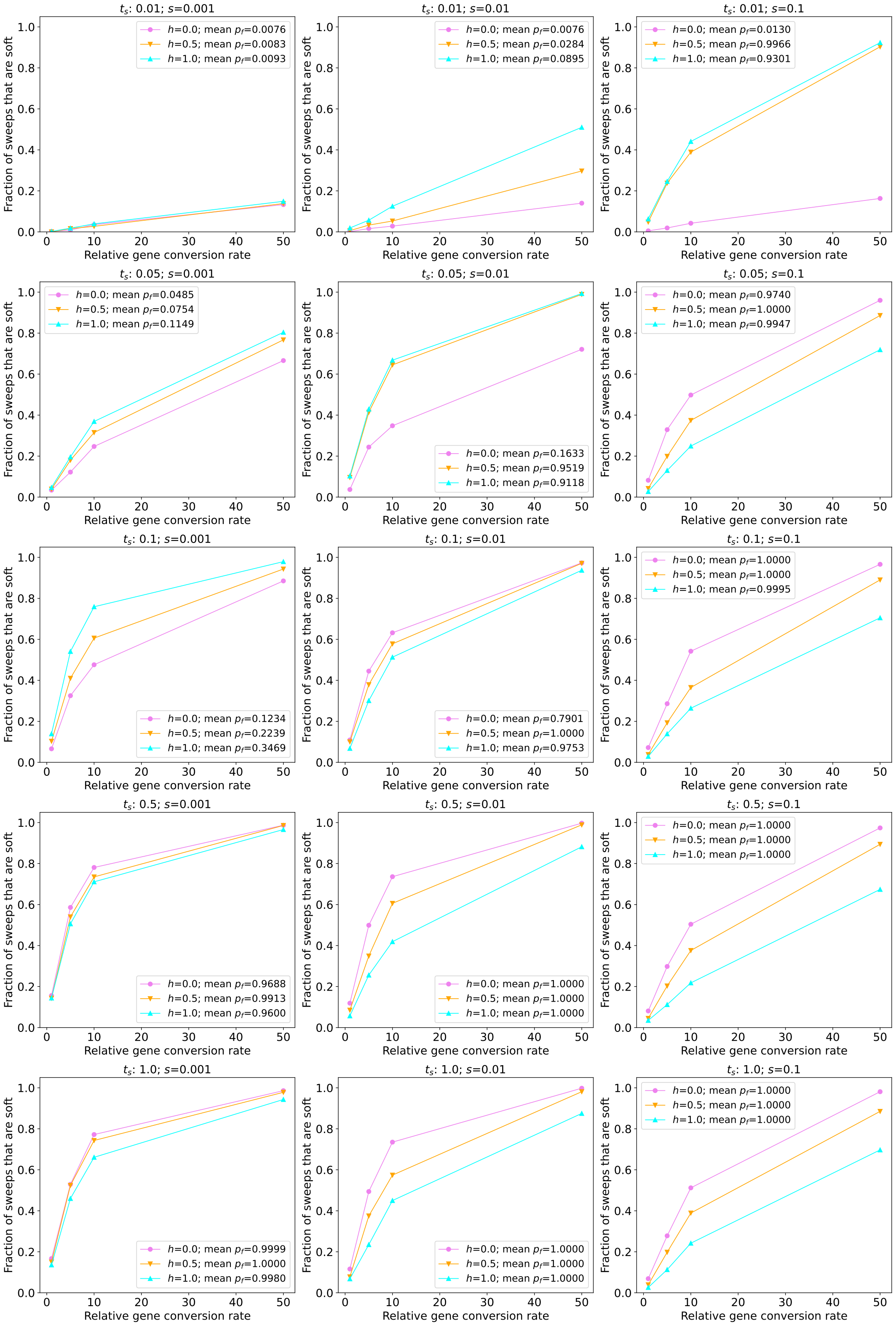

Supplementary Figure 1: The fraction of sweeps that are pseudo-soft for the constant-population size human simulations. The fraction of pseudo-soft sweeps at the time of sampling ($n = 200$ chromosomes) for a given combination of the selection coefficient (s) and the time since the start of the sweep (t_s) are shown in the appropriate panel, with the results for different dominance coefficients (h) shown as different colors as specified in the insets. Because for more recent sweeps there was often not sufficient time for the sweeping allele to reach fixation, the insets also show the average final frequency of the advantageous allele (p_f).

bioRxiv preprint doi: https://doi.org/10.1101/2023.12.05.570141; this version posted December 5, 2023. The copyright holder for this preprint (which was not certified by peer review) is the author/funder, who has granted bioRxiv a license to display the preprint in perpetuity. It is made available under aCC-BY 4.0 International license.

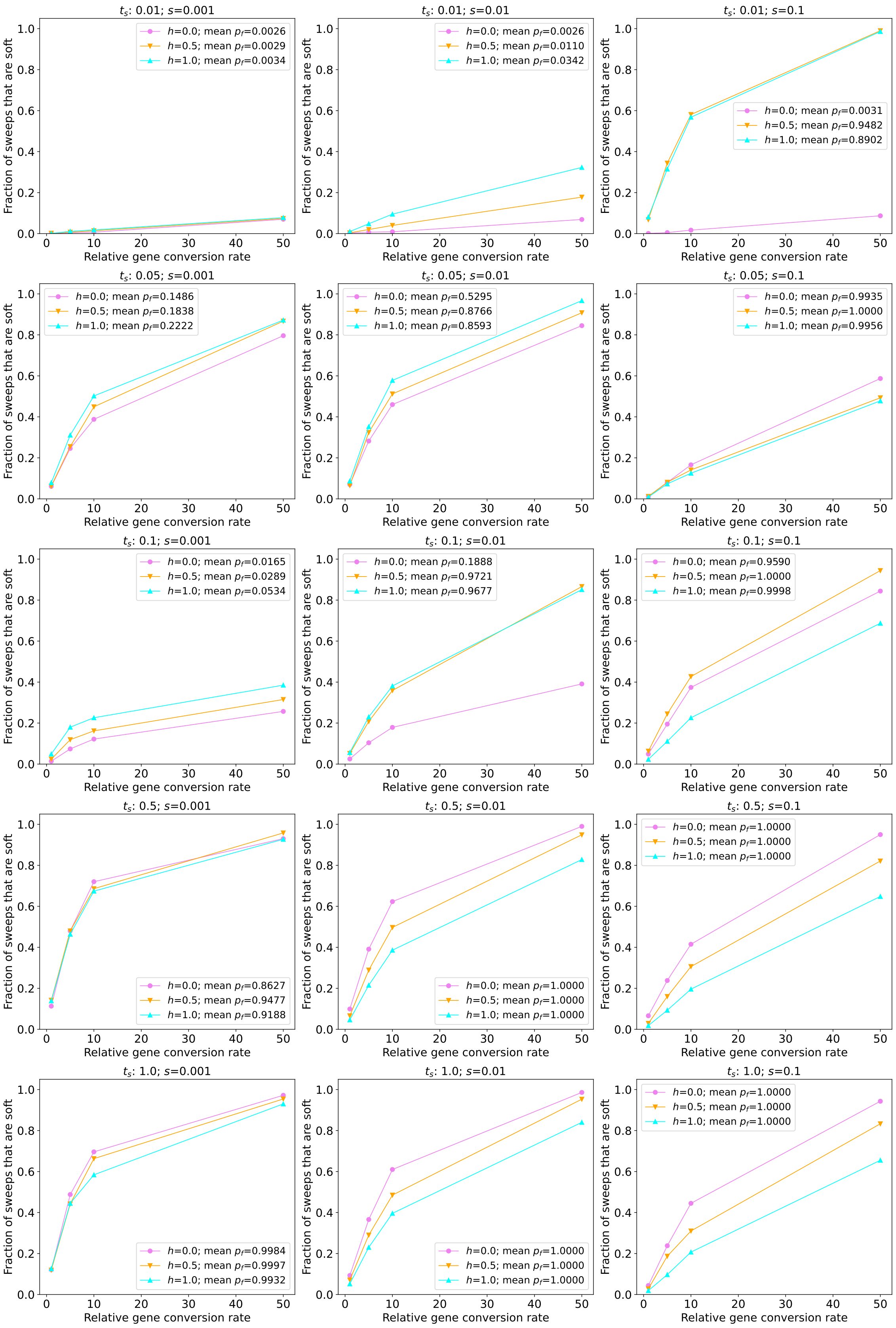

Supplementary Figure 2: The fraction of sweeps that are pseudo-soft for simulations under the human EUR model. The fraction of pseudo-soft sweeps at the time of sampling ($n = 200$ chromosomes) under a given combination of s and the time since the beginning of the sweep (t_s) are shown in the appropriate panel, with the results for different dominance coefficients (h) shown as different colors as specified in the inset. Because for more recent sweeps there was often not sufficient time for the sweeping allele to reach fixation, the insets also show the average final frequency of the advantageous allele (p_f).

bioRxiv preprint doi: https://doi.org/10.1101/2023.12.05.570141; this version posted December 5, 2023. The copyright holder for this preprint (which was not certified by peer review) is the author/funder, who has granted bioRxiv a license to display the preprint in perpetuity. It is made available under aCC-BY 4.0 International license.

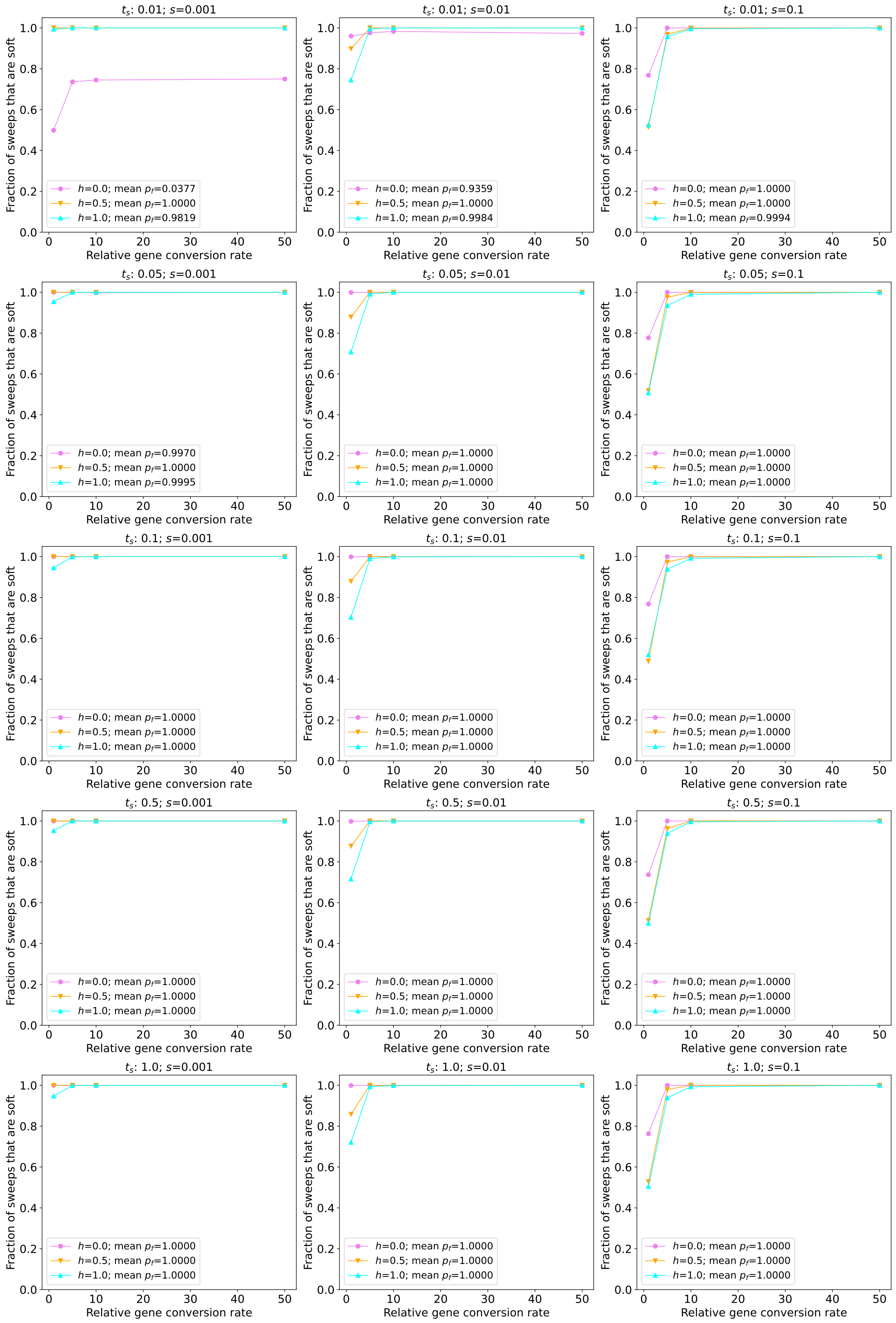

Supplementary Figure 3: The fraction of sweeps that are pseudo-soft for the constant-population size *D. melanogaster* simulations. The fraction of pseudo-soft sweeps at the time of sampling ($n = 200$ chromosomes) under a given combination of s and the time since the beginning of the sweep (t_s) are shown in the appropriate panel, with the results for different dominance coefficients (h) shown as different colors as specified in the insets. The average final frequency of the advantageous allele (p_f) for a given parameter combination is also shown in the inset. Note that for the most recent sweeps examined ($t_s = 0.01$), only 4% of recessive beneficial mutations with $s = 0.001$ had reached fixation at the time of sampling. However, for all other t_s values examined, approximately all sweeps reached fixation prior to sampling, and therefore the results are roughly identical to one another across values of t_s with the exception of the $s = 0.001$ and $h = 0.0$ case.

bioRxiv preprint doi: https://doi.org/10.1101/2023.12.05.570141; this version posted December 5, 2023. The copyright holder for this preprint (which was not certified by peer review) is the author/funder, who has granted bioRxiv a license to display the preprint in perpetuity. It is made available under aCC-BY 4.0 International license.

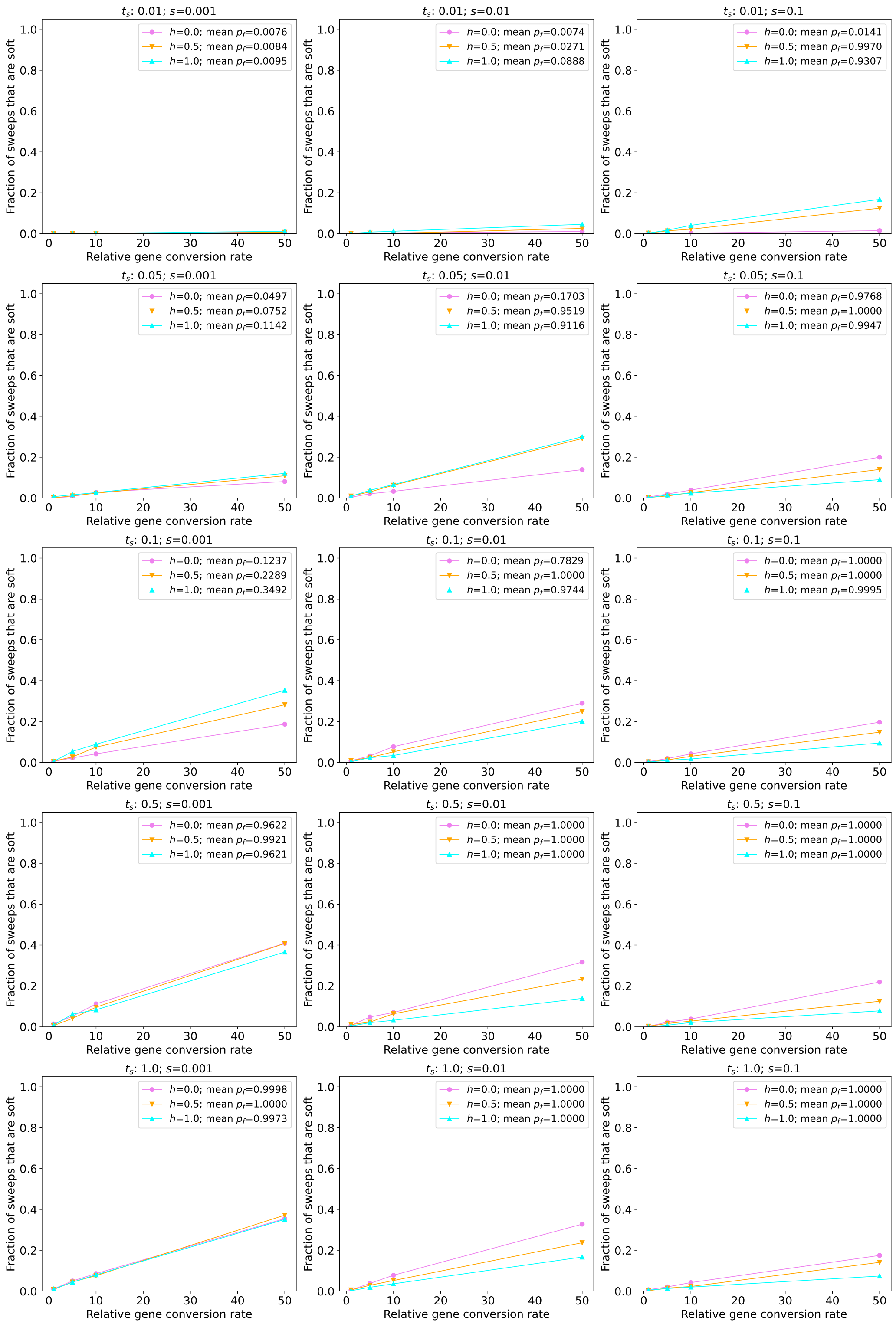

Supplementary Figure 4: The fraction of sweeps that are pseudo-soft for simulations under the *Arabidopsis* constant-size model. The fraction of pseudo-soft sweeps at the time of sampling ($n = 200$ chromosomes) under a given combination of s and the time since the beginning of the sweep (t_s) are shown in the appropriate panel, with the results for different dominance coefficients (h) shown as different colors as specified in the insets. Because for more recent sweeps there was often not sufficient time for the sweeping allele to reach fixation, the insets also show the average final frequency of the advantageous allele (p_f).

bioRxiv preprint doi: https://doi.org/10.1101/2023.12.05.570141; this version posted December 5, 2023. The copyright holder for this preprint (which was not certified by peer review) is the author/funder, who has granted bioRxiv a license to display the preprint in perpetuity. It is made available under aCC-BY 4.0 International license.

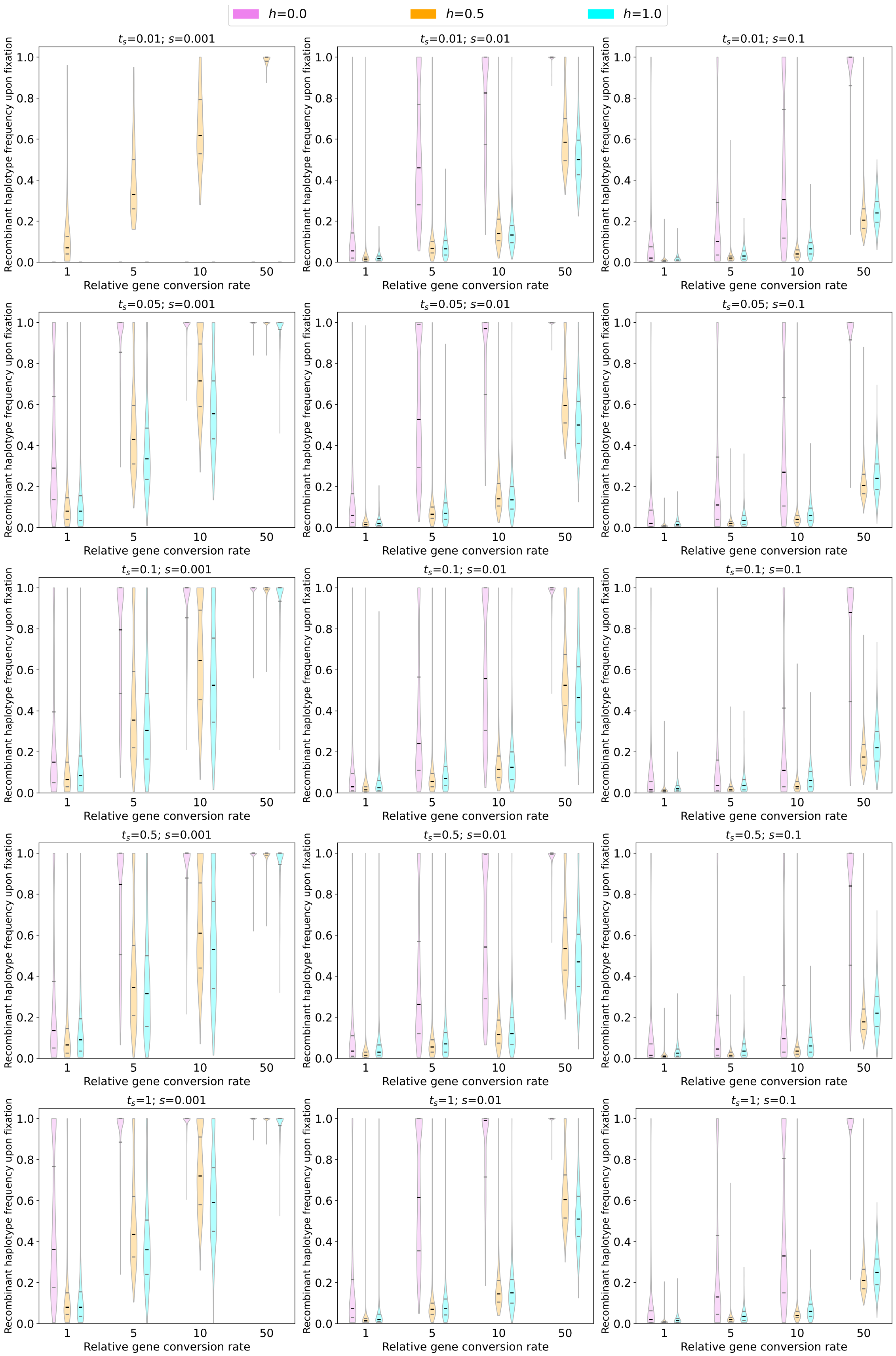

Supplementary Figure 5: The sample frequency of recombinant (aB) haplotypes upon fixation for each combination of s, h, and t_s under the *D. melanogaster* 3-epoch model (Sheehan and Song, 2016). Violin plots show the distribution of recombinant haplotype frequencies only for sweeps that had reached fixation at the time of sampling. For parameter combinations where no sweeps had fixed, no violins are shown.

bioRxiv preprint doi: https://doi.org/10.1101/2023.12.05.570141; this version posted December 5, 2023. The copyright holder for this preprint (which was not certified by peer review) is the author/funder, who has granted bioRxiv a license to display the preprint in perpetuity. It is made available under aCC-BY 4.0 International license.

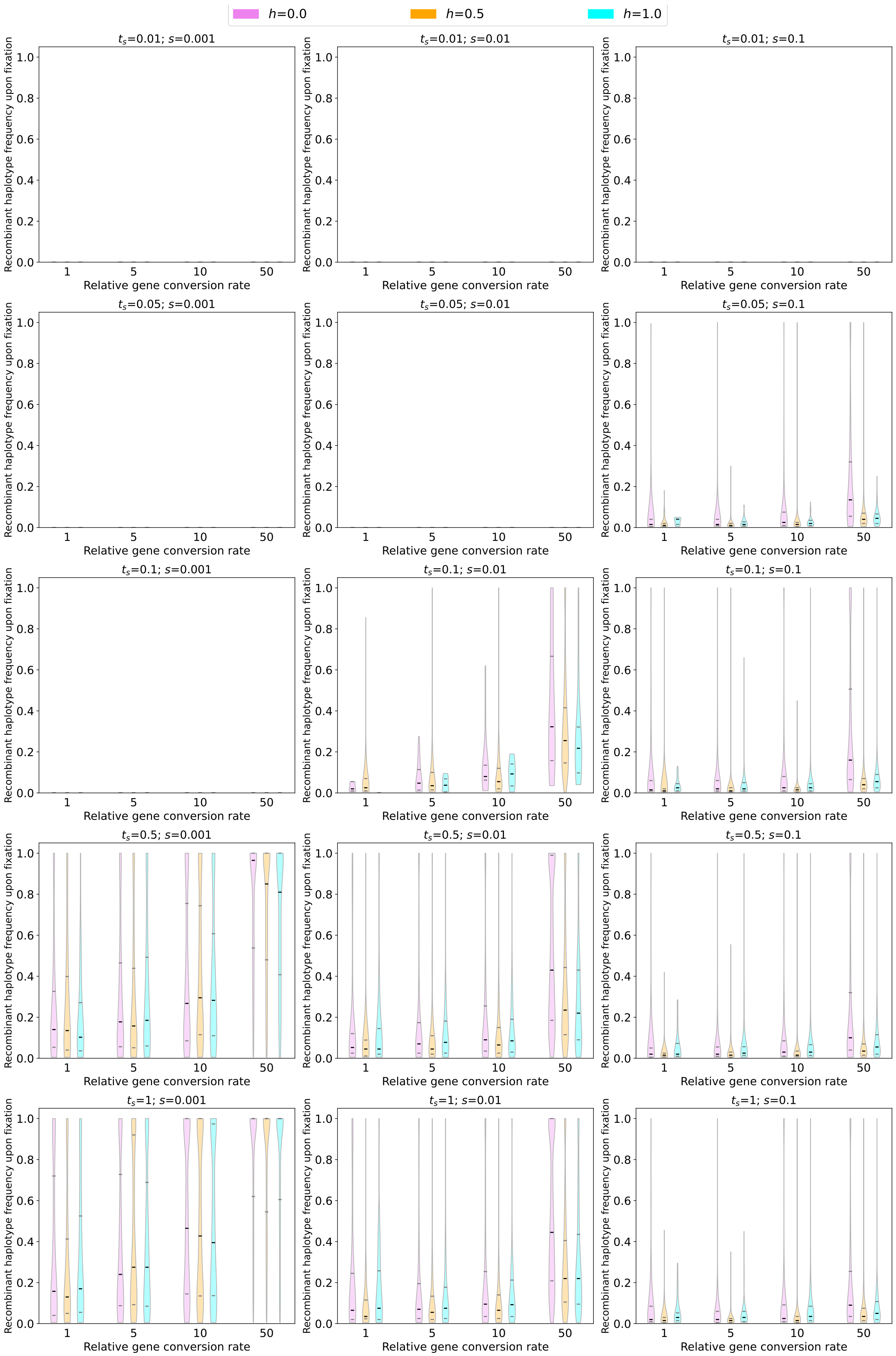

Supplementary Figure 6: The sample frequency of recombinant (aB) haplotypes upon fixation for each combination of s, h, and t_s under the human AFR model (Tennessen *et al.*, 2012). Violin plots show the distribution of recombinant haplotype frequencies only for sweeps that had reached fixation at the time of sampling. For parameter combinations where no sweeps had fixed, no violins are shown.

bioRxiv preprint doi: https://doi.org/10.1101/2023.12.05.570141; this version posted December 5, 2023. The copyright holder for this preprint (which was not certified by peer review) is the author/funder, who has granted bioRxiv a license to display the preprint in perpetuity. It is made available under aCC-BY 4.0 International license.

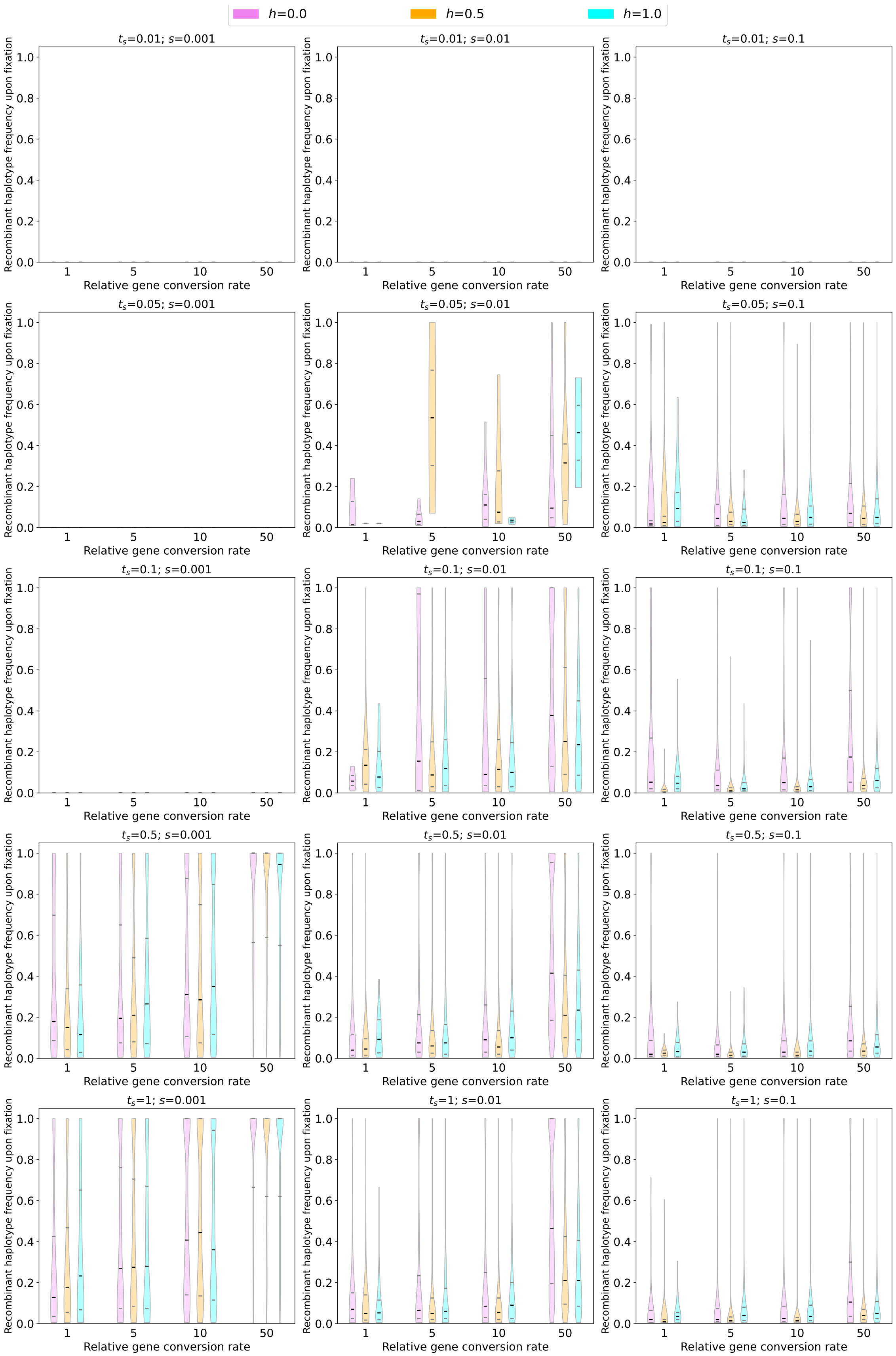

Supplementary Figure 7: The sample frequency of recombinant (aB) haplotypes upon fixation for each combination of s, h, and t_s under the human EUR model (Tennessen *et al.*, 2012). Violin plots show the distribution of recombinant haplotype frequencies only for sweeps that had reached fixation at the time of sampling. For parameter combinations where no sweeps had fixed, no violins are shown.

bioRxiv preprint doi: https://doi.org/10.1101/2023.12.05.570141; this version posted December 5, 2023. The copyright holder for this preprint (which was not certified by peer review) is the author/funder, who has granted bioRxiv a license to display the preprint in perpetuity. It is made available under aCC-BY 4.0 International license.

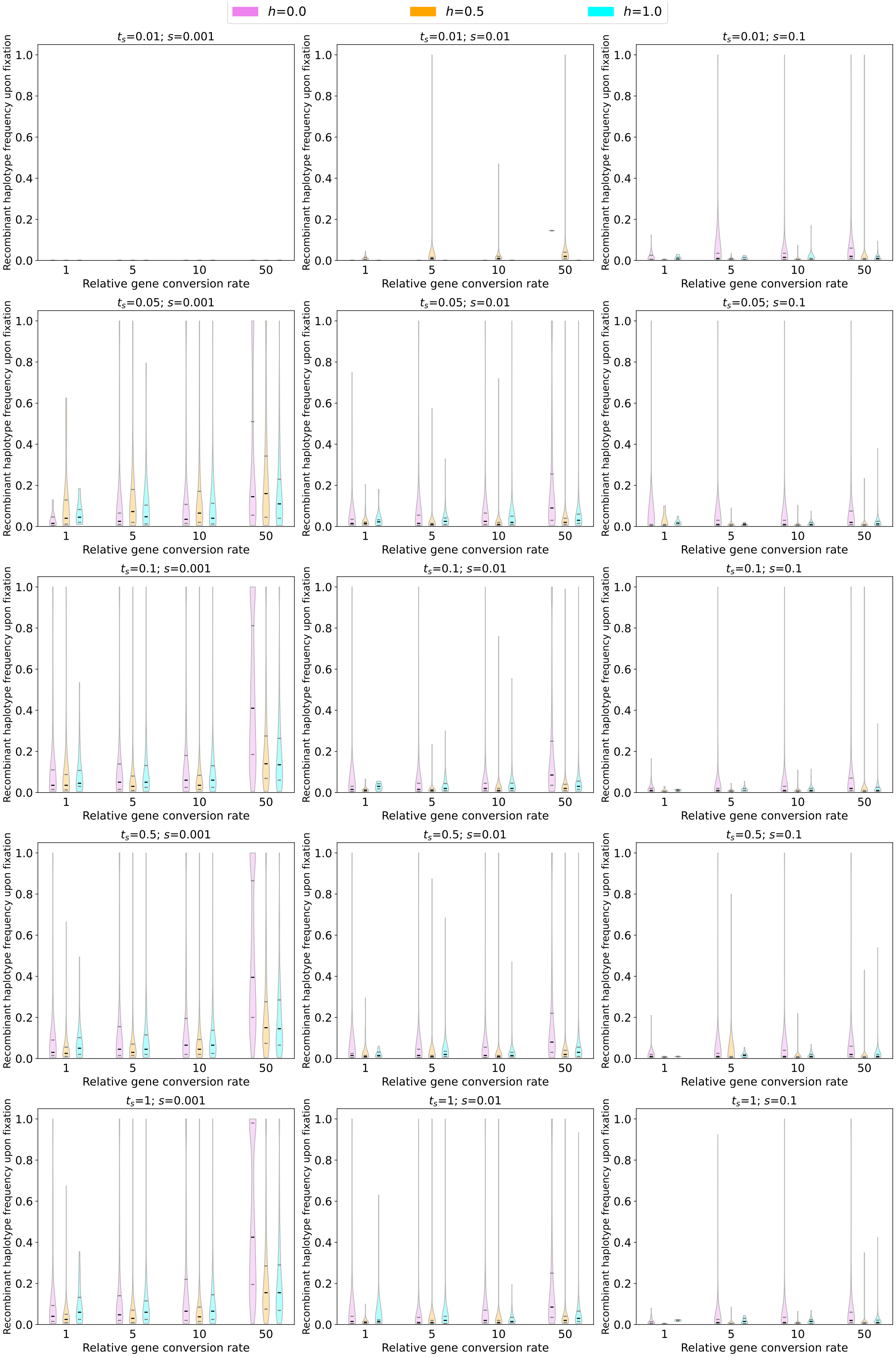

Supplementary Figure 8: The sample frequency of recombinant (aB) haplotypes upon fixation for each combination of s, h, and t_s under the *A. thaliana* 3-epoch model (Huber *et al.*, 2018). Violin plots show the distribution of recombinant haplotype frequencies only for sweeps that had reached fixation at the time of sampling. For parameter combinations where no sweeps had fixed, no violins are shown.
